# Prevalence of sarcopenia among Saudis and its association with lifestyle behaviors: Protocol for cross-sectional study

**DOI:** 10.1371/journal.pone.0271672

**Published:** 2022-08-02

**Authors:** Abdullah F. Alghannam, Alaa A. Almasud, Suliman A. Alghnam, Dalal S. Alharbi, Mohanad S. Aljubairi, Arwa S. Altalhi, Azad M. Jan, Shaima A. Alothman

**Affiliations:** 1 Lifestyle and Health Research Center, Health Sciences Research Center, Princess Nourah Bint Abdulrahman University, Riyadh, Saudi Arabia; 2 King Abdullah International Medical Research Center, King Abdulaziz Medical City, National Guard Health Affairs, Riyadh, Saudi Arabia; University of Mississippi, UNITED STATES

## Abstract

**Background:**

Sarcopenia is an age-related muscular disease manifesting as a loss of muscle function and mass–leads to detrimental consequences at both individual and community levels. Modifiable lifestyle factors (such as physical behaviors and nutritional habits) may be involved in sarcopenia etiology. European Working Group on Sarcopenia in Older Population (EWGOSP2) established a cut-off point for sarcopenia diagnosis based on the European population and they recommend the use of a regional normative population. However, no sufficient data on sarcopenia prevalence is presently available in Saudi Arabia. Therefore, this project aims to define appropriate reference values from healthy Saudi young adults (Phase I) and to investigate the prevalence of sarcopenia in Saudi Arabia (Phase II) and examine selected modifiable lifestyle correlates of sarcopenia (Phase I, II).

**Methods:**

The project will involve two phases. Phase I will include 1532 healthy Saudi young adults aged between 20–40 years. While, Phase II will include 1532 Saudi older adults aged ≥50 years. The study will measure vital signs, anthropometrics, muscle mass using bioelectrical impedance analysis and dual-energy X-ray absorptiometry, muscle strength using handgrip strength and maximal isometric strength, physical function using short physical performance battery, and 6-minute walk test to measure aerobic endurance. To explore the associations between lifestyle behaviors with sarcopenia indices, physical activity, sedentary behaviour and sleep will be evaluated subjectively using Global Physical Activity Questionnaire and Pittsburgh Sleep Quality Index and objectively via ActivPAL accelerometers. A three-day dietary food record will also be used to evaluate dietary intake. Additionally, EuroQOL five-dimension questionnaire will be utilized to assess health-related quality of life.

**Discussion:**

The study will have significant implications in recognizing the prevalence of sarcopenia in Saudi population, which will guide our future interventional studies aimed at early prevention and treatment of this disease.

## Introduction

Aging populations are a global phenomenon in the past few decades [1]. In Saudi Arabia, the 2019 census indicated that the elderly population ratio showed an increase by 59% compared to 2007 census [2]. Longer life expectancy is associated with an increased likelihood of morbidity and disability which might affect functional independence and quality of life [3]. Sarcopenia–defined as the age-related progressive loss of skeletal muscle mass and associated diminished muscular strength and functional capacity [4, 5]–poses a major challenge in the 21^st^ century [6]. Sarcopenia is officially recognized as a muscle disease with an ICD-10-MC diagnosis code [7]. The widely used definitions of sarcopenia is established by the European Working Group on Sarcopenia in Older Population (EWGSOP) and Asian Working Group on Sarcopenia (AWGS) [8, 9]. In 2018 and after exploring many aspects of sarcopenia, EWGSOP revised the original definition [10]. The sarcopenia diagnosis algorithm established by EWGSOP2 includes low muscle strength as a fundamental factor for sarcopenia diagnosis and it is confirmed by the presence of low muscle mass. The presence of low functional performance in addition to low muscle strength and mass is indicative of severe sarcopenia [10].

Sarcopenia has a high health and economic impact on both the individual and community levels. The presence of sarcopenia leads to several detrimental effects in terms of human health, including increased risk of falls and fractures [11], high level of dependence and long-term care usage [12, 13] and higher mortality rate [14], and decreased physical and cognitive capacities [15–17]. At the economic level, sarcopenia is significantly associated with higher health care cost and hospitalization; older adults with sarcopenia who are hospitalized have 5-fold higher health care cost than non-sarcopenic older adults [18]. A study in hospitalized patients reports that sarcopenia increases hospitalization by €1,240 (59% confidence interval (CI): €596–1,887) for patients aged 65 years and €721 (95% CI: €13–1,429) for patients aged ⩾65 years [19]. The aforementioned knowledge, therefore, clearly affirms the importance of global efforts in identifying, treating and preventing sarcopenia.

Sarcopenia appears to manifest earlier in life [20]. Noticeable reductions in skeletal muscle mass are apparent during the third decade of life and becomes more prominent by the late 50s [21]. While muscle strength reaches its peak in the 4^th^ decade and begins declining thereafter with aging [22]. Although an age-related disease, multiple other factors can trigger the development of sarcopenia, including modifiable lifestyle factors such as physical behaviors and nutrition [23, 24]. Many studies showed the positive effect of exercise and nutrition on sarcopenia [25–27]. These findings confirm the importance of early intervention to prevent or treat sarcopenia.

The prevalence of sarcopenia varies depending on the definition used, diagnostic criteria, cut-off points, and selection of assessment tool. Conservative estimates are ranged between 10% to12.9% in both males and females [28, 29]. However, the prevalence of sarcopenia in Saudi Arabia remains elusive. EWGOSP2 established a cut-off point for sarcopenia diagnosis based on the European population and they used healthy young adults from the same population as a normative reference (2 SD below the mean of reference value) and they recommend the use of a regional normative population as the age related muscle mass reduction significantly influenced by ethnicity [10, 30]. Only one study was conducted in Saudi Arabia to determine the reference values of sarcopenia in young Saudi men, however, this was not based on the current EWGOSP2 sarcopenia definition, in addition, no physical performance (functional) cut-off point was established [31]. To date, no cut-off points related to muscle mass, muscle function (strength and physical performance) are available for young Saudi females. Therefore, the present study aims to define appropriate reference values from healthy Saudi young adults (Phase I) and to investigate the prevalence of sarcopenia in Saudi Arabia (Phase II) and examine selected modifiable lifestyle correlates with sarcopenia (Phase I, II), as these influences may have a role in the development of sarcopenia much earlier in life than previously thought [5, 20].

## Methods

### Research aims and objectives

The objectives of this study will be differentiated based on the age group targeted into two phases. Phase I will include a sample with an age group range between 20 and 40 years old to obtain reference values for sarcopenia indices among Saudis. Phase II will include a sample with an age group ≥50 to determine sarcopenia prevalence in Saudi Arabia. The main objectives for both phases are described in (Table 1).

**Table 1:** Objectives of the study.

Phase I	Phase II
(1) Investigating muscle mass and muscle function in healthy Saudi young adults	(1) Investigating muscle mass, muscle function in Saudi older adults
(2) Exploring the associations between lifestyle behaviors for instance, physical activity, sedentary behavior, nutrition, and sleep with sarcopenia indices in healthy Saudi young adults	(2) Exploring the associations between lifestyle behaviors including physical activity, sedentary behavior, nutrition, and sleep with sarcopenia indices in Saudi older adults
(3) Examining the agreement between dual-energy x-ray absorptiometry (DXA) and bioelectrical impudence analysis (BIA) in assessing lean mass in healthy young Saudis.	(3) Examining the agreement between DXA and BIA in assessing lean mass in older Saudi adults.

### Study design and setting

This analytic cross-sectional study will be conducted at Lifestyle and Health Research Center, Health Sciences Research Center at Princess Nourah bint Abdulrahman University in Riyadh City. This study will be conducted over a period of 24 months, from May 2021 to May 2023 (both phases).

### Recruitment

A simple random sampling method will be used for recruitment, in which participants will be recruited from the local community via word-of-mouth and advertisement (including social media, announcement boards, events at primary care clinics, and associations related to the elderly). Upon contacting researchers assigned to the study, participants will be provided with a participant information sheet detailing the study aims and requirements. Participants who express their interest in the study will be assessed for eligibility and provide an opportunity to discuss any details or requirements related to the study’s procedures through telephone-based interviews. Medical health and socio-demographics sheets will be sent electronically via research electronic data capture software (REDCap) [32, 33]. If a participant meets the study inclusion criteria, he/she will be invited to take part in the study. Phase I and II of the study will have a similar methodology except for the inclusion/exclusion criteria (detailed below).

### Selection criteria

The eligibility criteria for participants in both phases are presented in (Table 2).

**Table 2:** Selection criteria.

	Phase I	Phase II
Inclusion criteria	• Volunteers aged between 20 and 40 years old • Cleared from cardiovascular, metabolic, renal or malignant diseases • Non-pregnant or breastfeeding females	• Volunteers aged ≥50 years • Able to walk independently for at least 50 meters • Free from malignant diseases.
Exclusion criteria	• Physical or mental incapacity to follow study procedures and assessments • Presence of congenital muscular or neurological diseases/deformity • Presence of any metal implants or pacemaker • Reported use of substances or other conditions or behavior which may pose an undue personal risk to participants or introduce bias into the study	• Physical or mental incapacity to follow study procedures and assessments • Inability to walk independently for 50 meters • Congenital muscular or neurological diseases/deformity • Presence of any metal implants or pacemaker • Reported use of substances or other conditions or behavior which may pose undue personal risk to participants or introduce bias into the study

### Assessment of sarcopenia

EWGSOP2 definition of sarcopenia will be applied for this study. The cut-off values for determining sarcopenia will be based on EWGSOP2 criteria (Table 3) [10]. While in Phase II the cut-off values will be based on the normative data obtained from Phase I. Bland and Altman plots will be used to determine absolute bias between DXA and BIA using 95% limits of agreement.

**Table 3:** Sarcopenia cut-off values.

Phase I	Phase II
• EWGSOP2 sarcopenia cut-off value for low strength by handgrip strength **Male, <27 kg; female, <16 kg** • EWGSOP2 sarcopenia cut-off values for low muscle quantity **Male, <7.0 kg/m**^**2**^**; female, <5.5 kg/m**^**2**^ EWGSOP2 sarcopenia cut-off values for low performance (by Short physical performance battery (SPPB) **Male and female ≤8 points**	The cut-off values will be based on the normative data obtained from (Phase I)

### Study protocol

**Fig 1:**
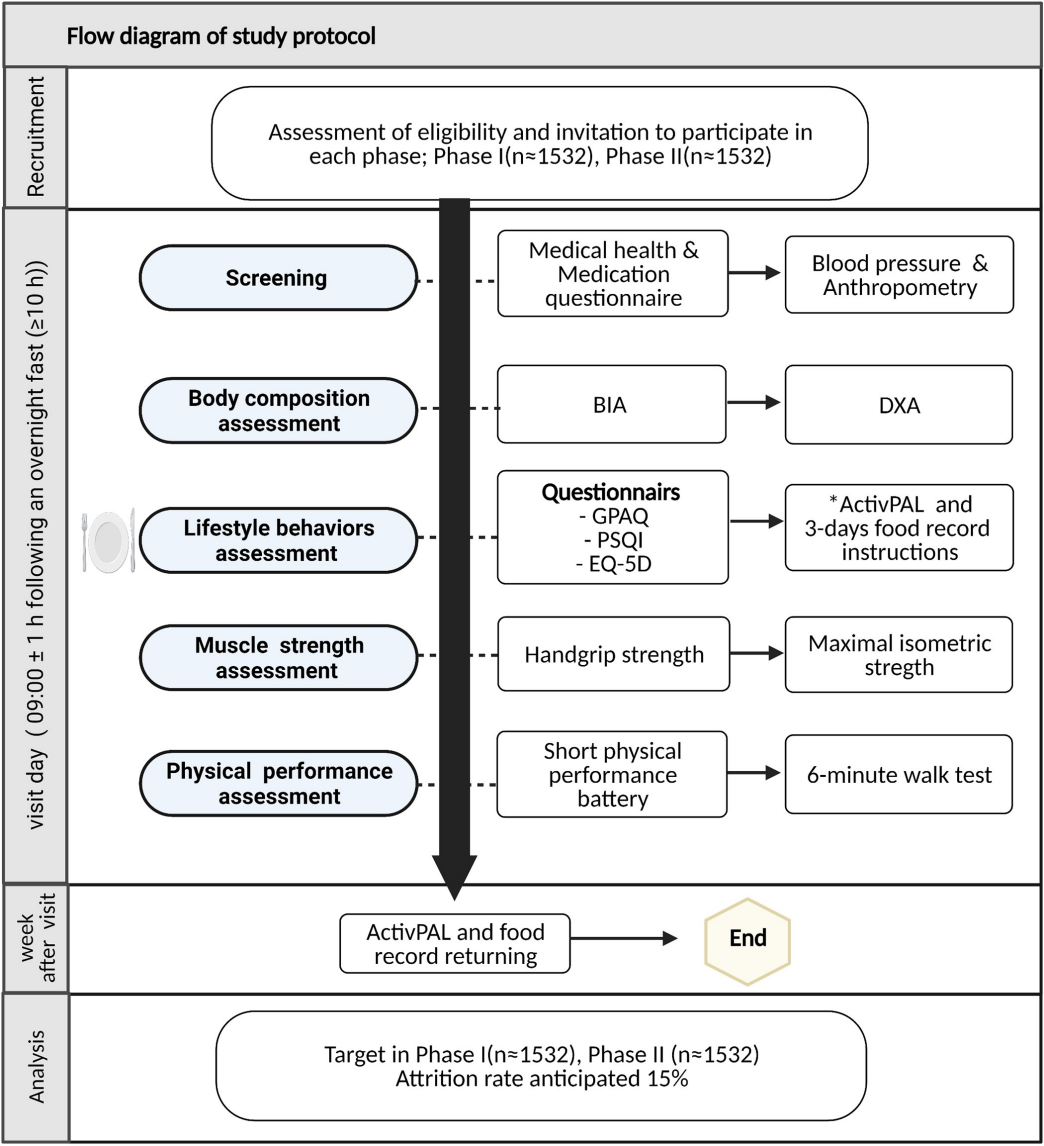
Flow diagram of study protocol. BIA, Bioelectrical impedance analysis; DXA, Dual-energy X-ray absorptiometry; GPAQ, Global Physical Activity Questionnaire; PSQI, Pittsburgh Sleep Quality Index; EQ-5D, EuroQOL five-dimension questionnaire. * activity monitor small device worn that uses information about static and dynamic acceleration. A healthy snack will be given to the participants while they fill the questionnaires.

On visiting day, each participant will arrive at the laboratory at 09:00±1 h following an overnight fast (≥10h), wearing comfortable outfit without any metal accessories. Participants will be required to bring their medication if they are on any type of medication. Upon arrival and after filling the consent form, participants will undergo a brief medical health and medication questionnaires, then post-void body mass, standing height, waist and hip circumference, and blood pressure will be measured. Next, participants will undergo body composition assessment using BIA and DXA, respectively. Thereafter, a snack will be given while they complete subjective questionnaires relating to their physical activity, sedentary behavior, sleep and quality of life. Thereafter, ActivPAL wearing and a three-day food record filling instruction will be given to the participants. 30 minutes after a snack, blood glucose will be measured. Handgrip strength will then be assessed, followed by maximal isometric knee extensions to assess upper and lower limb strength, respectively. This will be followed by a functional test (SPPB). Lastly, participants will undergo a 6-minute walk test. Week after, ActivPAL and 3-days food record will be returned. Ambient temperature and humidity will be monitored and recorded at 60-minute intervals during each laboratory visit. Study protocol process is described in sequence in (Fig 1).

### Outcome measures

#### Sociodemographic data

Age, sex, marital status, educational level, employment status and position, phone number, and another contact number in case of emergency will be taken from participants.

#### Vital signs

Blood pressure will be measured before the beginning of testing, while glucose level will be assessed after a snack. Blood pressure will be measured via (Connex® Spot Monitor, Welch Allyn, USA), participants will rest on a chair for at least 5 minutes before measurement. Further, blood glucose level will be measured via a finger stick using a glucometer (ACCU-CHEK PERFORMA, Roche, Germany). Participants with uncontrolled hypertension (>190/100 mmHg) and/or glucose level below 70 mg/dL or above 300 mg/dL will be excluded from the study to maintain their safety during physical function testing.

#### Anthropometric measures

Height to the nearest 0.5 cm and post-void body mass to the nearest 0.1 kg will be taken from participants with minimal clothing and without shoes via (Seca274, Seca, Germany). Body Mass Index (BMI) (kg·m^2^) will be measured based on height and weight. Further, waist circumference to the nearest 0.1 cm at the umbilicus will be measured using non-stretchable measuring tape. From standing, participants will be instructed to exhale while the examiner takes the measurements. Then, hip circumference to the nearest 0.1 cm at widest gluteal girth will be measured from standing using non-stretchable measuring tape. Body Shape Index (BSI) will also be calculated as BSI = Waist circumference / ((BMI ^2/3^) x (height ^1/2^)) [34].

#### Body composition measures

*Bioelectrical impedance analysis*. BIA model (mBCA 515, Seca, Germany) with a constant high frequency current 100 mA at a frequency of 50 kHz and an electrode system will be designed to measure the body composition in segmental parts from the whole body, including arms, legs, and the trunk area. Therefore, fat mass (FM), fat-free mass (FFM), the predicted muscle mass of the appendicular fractions, and appendicular skeletal muscle mass (ASM), could be estimated by the sum of each segment except for the ‘‘trunk part” as previously reported [31]. From these measures (ASM/height^2^), (ASM/ weight) and appendicular skeletal muscle mass index (ASMI) will be calculated. Prior to the test, the participants will be asked to remove shoes and metals and ensure they have dry hands and feet. Then, they will be asked to stand in the middle of the weighting platform with feet on marked circles and hold the handles of the machine appropriate to their height (upper extremity not touching body). After that, measurement will be taken and the results will be printed directly at the end of the test.

*Dual-energy X-ray absorptiometry*. DXA is a feasible, accurate, safe instrument for measuring muscle mass [35]. Body composition (lean mass and fat mass) will be assessed using DXA (Lunar iDXA, GE Healthcare, USA) scan through enCORE software platform. Daily prior to test or if room temperature changed more than 5°C quality assurance procedure will be done using phantom block to assess functionality assurance and precision of the densitometer. The coefficient of variation for DXA scan lower than 2% will be accepted. A total-body DXA scan will be done to all participants by a single trained technician, according to the manufacturer’s instructions (GE Healthcare, USA). The test will be performed to all participants in the same time and in fasting status to ensure similar hydration level. However, hydration status will not be measured objectively. Before starting the test, subjects will be requested to remove all metal accessories such as (jewelry, belt) and will be asked to put the phone on a silent mode. Then, they will lie in the middle of the table bed in a natural position all body parts must be included in the scanning spectrum. Upper extremity beside but not touching the body, knees and ankles are close together and stabilized by velcro straps. Appendicular lean mass (ALM) will be calculated as the sum of lean mass from arms and legs. Fat mass, lean mass, and ALM are going to be presented as absolute values (in kg) and as a percentage of body weight. ALM adjusted for height is going to be calculated as lean mass divided by squared height (kg/m^2^). The result will be printed directly at the end of test.

#### Lifestyle behaviors measurements

*Physical activity and sedentary behavior*. Physical activity (PA) and sedentary behavior (SB) will be assessed subjectively and objectively for all participants. Global Physical Activity Questionnaire (GPAQ), a modified version of the International Physical Activity Questionnaire (IPAQ) developed in 2002 by the World Health Organization (WHO) [36] will be used to subjectively estimate an individual’s PA level. The Arabic version of the questionnaire had been tested for its validity and reliability [37]. It is a self-reported questionnaire that collects information on participant PA frequency (time/week), duration (min), and intensity (light, moderate, and vigorous) during a typical day [38]. GPAQ consists of 16 questions designed into 3 main domains (work, transport, and leisure time) and one additional question related to SB asking about the total time spent sitting per day. Total PA is then calculated by the sum of the total metabolic equivalents (MET) minutes of activity computed for each domain [38]. Additionally, participants will be classified to have low, moderate, or high level of PA.

Objective assessment will be obtained using ActivPAL monitor (PAL Technologies Ltd., UK).

ActivPAL monitor is a small device worn on the thigh (one third of the way between hip and knee as manufacture instructions) that uses information about static and dynamic acceleration to identify body postures as lying, sitting, standing or stepping and estimate energy expenditure as metabolic equivalents [39]. Using ActivPAL software takes the recorded signal from the thigh sensor and analyse it via algorithms to produce a record of the activity events. Participants will be asked to wear the ActivPAL monitor for 7 consecutive days, removing the monitor only when they are fully dipped in water and record non-wear time in recording sheet. In addition, participants will be asked to record their sleep in a sleep diary to account for sleeping time. For the daily data to be included in the analysis, participants need to wear the ActivPAL for at least 10-hours of waking hours on at least four out of seven days.

*Sleep assessment*. Sleep quality will be assessed by completing the Pittsburgh Sleep Quality Index (PSQI). It is a self-rated questionnaire that assesses sleep quality and disturbances in a one-month time interval. The Arabic version of the questionnaire had been tested for its validity and reliability [40]. It consists of 19 items creating seven components (subjective sleep quality, sleep latency, sleep duration, habitual sleep efficiency, sleep disturbances, use of sleeping medication, and daytime dysfunction). The global score (range between 0–21) obtained from the sum of scores for these seven components; Poor sleepers have ≥ 5 scores as a cut-off global score [41]. Further, sleep duration will be assessed both objectively and subjectively using the above-mentioned ActivPAL monitor and sleep diary.

*Quality of life assessment*. EuroQOL five-dimension questionnaire (EQ-5D) will be used to assess quality of life. It is a comprehensive multidimensional self-reported questionnaire that integrates physical, cognitive and social well-being [42]. It consists of two parts, the first part (descriptive system) assesses health in five domains: self-care, mobility, usual activities, pain/discomfort, and anxiety/depression. Each of which has three level of response (no problems, some problems, and extreme problems/unable). The second part consists of visual analogues scale (VAS) in which participant rate his perceived health form 0 (the worst imaginable health) to 100 (the best imaginable health) [43]. Valid and reliable Arabic version for this instrument will be used in this study [44, 45].

*Nutrition assessment*. Usual food intake will be assessed using three days of dietary food record; a self-reported record has been shown to be valid and reliable to assess nutrition [46]. With clear instructions, participants will be asked to record their normal dietary habits and supplements (two weekdays and one weekend day) in the week after laboratory visit. After that, the record will be reviewed by a trained interviewer through a phone call. Data will then be analyzed using nutrition analysis software (Nutritics LTD, Ireland). The average intake of the three days will be used as the usual food intake. Glycemic index (GI) and glycemic load (GL) will be calculated using GI values from Foster-Powell 2002 and Atkinson 2008 [47, 48]. The GI will be calculated by multiplying GI value of each food item by the amount of carbohydrates in that food item (g) divided by the total carbohydrates (g) [49]. The GL will be calculated by multiplying GI value of each food item by the amount of carbohydrates in that food item (g) divided by 100. The GI and GL values of each participant’s diet will be calculated as the sum of the GI and GL for each food item. Diets with GI values of ≥70, between 69–54, and ≤55 are classified as high, medium, and low GI, respectively [47, 48]. Diets with GL values of ≥120, between 80–119, and <80 are classified as high, medium, and low GL, respectively [50, 51].

#### Muscle strength measurements

*Handgrip strength*. Handgrip strength (HGS) will be measured using a digital hand dynamometer (Digital grip strength dynamometer, T.K.K 5401, Takei Scientific Instruments Co., Ltd., Japan) to assess muscle strength. Absolute HGS (kg) and relative (HGS (kg)/body mass (kg)), will be recorded for each participant. The test will be performed in a standing position with the forearm away from the body at the level of the thigh. After size adjustment, participants will be asked to exert maximum grip strength three times with both dominant and non-dominant hands, allowing at least 30-seconds of resting interval as previously reported [52].

*Maximal isometric strength*. Maximal isometric strength will be measured using Humac NORM dynamometer (CSMi, Stoughton, USA) to assess muscle strength in knee extensors and flexors [53]. Prior to the test, participants will perform a five-minute warm-up on a bicycle ergometer (Ergomedic 828E, Monark, Sweden), at an intensity of 40 watts. Participants will be seated in the testing chair with a hip flexion angle of 110°. The back of the knee joint will be placed on the edge of the seat with an angle of -60° from anatomical zero (180°), which has been established to be the angle of maximal isometric force generation [54]. Velcro strap will be used to attach the distal shin pad of the dynamometer 4-5cm above the medial malleolus. A four-point belt and Velcro straps will be used to reduce extraneous movement during contractions of the chest, pelvis and thigh. The lateral femoral condyle will be aligned with the dynamometer rotational axis. Participants will be instructed to perform three maximal voluntary isometric contractions (MVC) of the knee extensor separated by one minute of stationary rest with unbaled gravity. After receiving a 5-second count down, they will be instructed to steadily produce their maximal force rapidly and maintaining that force for 3–4 seconds. Thereafter, participants will receive 2–3 minutes of stationary rest to prepare the set-up of the protocol to measure MVC of the knee flexors. The distal shin pad will be replaced along the posterior portion of the triceps surae; 4-5cm above the medial malleolus. Participants will then be instructed to repeat the same procedure whilst kicking back (flexion) in the sagittal plane. Visual feedback on a computer screen of the immediate dynamometer torque will be displayed to participants to illustrate the type of contraction required and provide encouragement. MVC will be analysed for peak torque using Humac Norm Isokinetic Software System. The analysis tool will be used to place the cursor at the beginning of the first visible rise in the torque time trace and moved across until it met a horizontal phase of contraction. The software will then show both times to peak torque in seconds (s) as well as peak torque in newton meters (N·m). At the end of the test result will be printed.

#### Physical performance measurements

*Short physical performance battery*. Short physical performance battery will be used to assess physical function, due to its high validity, reliability, cost-effectiveness and ability to detect changes over time [55, 56]. The test evaluates lower extremity function consists of three sub-scales to evaluate balance (side by side stand, semi-tandem stand and tandem stand), speed (gait speed) and force (chair stand test) [57]. Each sub-scale is allocated a score between 0–4 with 0 being “unable to complete the task” and 4 being the “highest level of functional performance”. With safety consideration and under supervision of the examiner, the participant will be asked to stand with feet close to each other (side by side stand), then they will stand with one heel against side of big toe of the other foot, followed by standing with the heel of one foot against the toes of other foot (holding 10 sec for each position). Thereafter, the participants will be asked to walk 4-m (using their walking aids if available) with normal speed two times and the highest record out of two repetitions will be taken. The last test will be performed by 5 times sit to stand with arm crossed the body at fastest speed. Scores from each sub-scale are then added to create a global score between 0–12. A cut-off point of <8 points is used to identify poor physical function [58, 59].

*Aerobic endurance*. Participants’ aerobic endurance capacity will be measured via a 6-minute walk test (6MWT)–a commonly used, valid and reliable test to assess cardiovascular endurance in different populations [60]. A 32-m hallway with a smooth surface and chair at one end will be prepared prior to test. At the beginning the participant will be sitting in a chair near the starting point. Once timing is started, participant will walk with normal speed (back and forth) for 6-minutes and the assessor will walk behind them for safety and to count laps. They can use walking aid (the type of walking device and/or bracing must be documented). If they require assistance, only the minimum amount of assistance required for completing the task will be provided, level of physical assistance will be documented using an ordinal (1–7) point scale where, 1 means “total assistance needed” and 7 means “totally independent”. Distance covered in six minutes will be calculated by multiplying number of total laps by 32-m.

### Statistical analysis

#### Sample size calculation

Sample size calculated using the following formula: n = Z2P(1−P)d2; Where n is the sample size, Z is corresponding to level of confidence, P is expected prevalence and d is precision [61]. *A Priori* sample size was conducted based on a sample proportion within 0.5 (i.e., proportion that yields maximal possible sample size required) of the population proportion with a 95% confidence level. Assuming type I error of 1% and a precision of 5% the sample size required for each phase will be 1332 participants. Therefore, 1532 male and female participants will be recruited to account for an anticipated 15% non-completion rate.

#### Data analysis

Descriptive statistics will be used to characterize the demographics and measured variables of the participants. Normality will be assessed for continuous variables using the Shapiro-Wilk test. The prevalence of sarcopenia will be calculated. Parametric data will be reported as mean ± standard deviation (SD), or a non-parametric equivalent (e.g., median and inter-quartile range) data deemed non-normally distributed. Comparisons between two means will be analyzed by an independent t-test or a non-parametric equivalent (i.e., Mann-Whitney U test) to compare medians.

Multivariate logistic regression will be used to examine the association between sarcopenia and lifestyle-related factors (physical activity, sedentary behavior, sleep and nutrition) with odds ratios and 95% confidence intervals (95% CI). A separate analysis will also be performed with low muscle mass, low grip strength and low functional performance as dependent variables. Age and BMI will be included in the regression analysis, because of their established associations with grip strength and muscle mass [62]. The primary outcome measures will be sarcopenia indices: skeletal muscle mass, muscle strength and functional performance. Missing data will be evaluated and addressed using appropriate statistical methods, such as multiple imputation. Statistical procedures will be performed formed using commercially available software (Prism 9.0, GraphPad, USA) and significance will be set at an alpha level of ≤ 0.05.

#### Data management and monitoring

Data will be entered on and stored at a secure, password-protected computer. As part of the data protection and confidentiality, all participants will be assigned unique coding identities. The database will be managed by the investigators and access will be limited to individuals deemed appropriate.

## Discussion

Knowledge of sarcopenia prevalence in Saudi Arabia will help in addressing its regional impact on human health and health care cost through: (1) defining Saudi reference values fundamental in diagnosing, treating and preventing sarcopenia; (2) determining cut-off points related to muscle mass, strength and physical performance and thus guiding future studies in Saudi Arabia; (3) understanding the modifiable lifestyle factors in a young age group and therefore assisting in future studies aiming to prevent or delay sarcopenia. Ultimately, this research project addresses a global health topic of utmost importance, with an unmet medical need in Saudi Arabia.
